# Core circadian clock gene expression in human dental pulp‐derived cells in response to L‐mimosine, hypoxia and echinomycin

**DOI:** 10.1111/eos.12535

**Published:** 2018-07-13

**Authors:** Klara Janjić, Christoph Kurzmann, Andreas Moritz, Hermann Agis

**Affiliations:** ^1^ Department of Conservative Dentistry and Periodontology University Clinic of Dentistry Medical University of Vienna Vienna Austria; ^2^ Austrian Cluster for Tissue Regeneration Vienna Austria

**Keywords:** chronobiology phenomena, endodontics, fibroblasts, hypoxia mimetic agents, pulp regeneration

## Abstract

Core circadian clock genes set the pace for a wide range of physiological functions, including regeneration. The role of these genes and their regulation in the dental pulp, in particular under hypoxic conditions, is unknown. Here we investigated if core clock genes are expressed in human dental pulp‐derived cells (DPC) and if their expression is modulated by the hypoxia mimetic agent, L‐mimosine (L‐MIM), hypoxia or echinomycin. Dental pulp‐derived cells in monolayers and spheroids were treated with L‐MIM, hypoxia or echinomycin. mRNA levels of the core circadian clock genes were analysed using quantitative PCR (qPCR) and their protein levels were analysed by western blot. All core clock genes and proteins were produced in DPC monolayer and spheroid cultures. The expression of cryptochrome circadian regulators and period circadian regulators was reduced by L‐MIM, hypoxia and echinomycin at mRNA, but not at protein levels. Time course experiments indicated that modulations were based on alterations in overall mRNA levels of core circadian clock genes. Our results suggest a potential role of the core circadian clock in the response of dental pulp to hypoxia. Future studies need to consider that regulation of the core circadian clock at mRNA levels might not be paralleled by modulation of protein levels.

The development of novel strategies for tissue regeneration should be based on the understanding of underlying biological functions. An optimal adaption to changing environments has been one of the key drivers of evolution. Organisms are able to synchronize a variety of molecular and physiological functions, such as behaviour, reproduction or cell division, with steady cycles of stimuli that come from the environment. These stimuli – the *zeitgebers* – can be temperature, food or the daily light–dark cycle, if they act as cues to synchronize or entrain molecular clocks. Molecular clocks play a crucial role behind these mechanisms, allowing various aspects of life to be improved and increasing the chance of survival for many species 1. But how do molecular clocks tick? The most studied molecular clock is the circadian clock, which is mainly driven by daily light–dark cycles, resulting in 24 h oscillation of certain clock genes. When a periodically recurring environmental stimulus (input) is registered, it entrains the central circadian clock (oscillator) in the suprachiasmatic nuclei of the brain 2. There, the central circadian clock transmits external stimuli into a rhythmical endogenous pathway that controls a physiological function (output). The molecular mechanism in the central circadian clock is based on transcriptional/translational feedback loops between core clock genes. In mammals, the proteins circadian locomotor output cycles kaput (CLOCK) and aryl hydrocarbon receptor nuclear translocator‐like protein 1 (BMAL1) form a heterodimer. They act as transcription factors when binding to the E‐boxes in the promotor regions of period circadian regulator (*PER1*,* PER2*,* PER3*) and cryptochrome circadian regulator (*CRY1*,* CRY2*) genes. Period circadian regulator (PER1, PER2, PER3) and cryptochrome circadian regulator (CRY1, CRY2) proteins accumulate in the cytoplasm and dimerize. The heterodimer is then translocated to the nucleus where it inhibits expression of *CLOCK* and *BMAL1* genes and with this starts a negative feedback loop on its own gene expression. This feedback cycle repeats every 24 h 3. Each of the core clock genes has a specific function in regulating period length, rhythm persistence and amplitude of oscillations. These functions have been determined in numerous in vivo loss‐of‐function experiments in which disruption of circadian rhythm can lead to severe pathologies 4. Core clock mechanisms are also found in almost every peripheral tissue, including the core clock genes and clock‐controlled genes 5.

Although a role of molecular clocks has been proposed in oral tissue and tooth development, the existence and function of the core clock genes in the dental pulp is currently unclear 6. As molecular clocks regulate a variety of different physiological and cell biological processes, it is likely that they also have an influence on regeneration. An implication in governing tissue regeneration has been reported for example, in lungs 7, skeletal muscles 8, vascular system 9, hair follicles 10, intestine 11 and liver 12. Understanding the principles of molecular clock mechanisms in the dental pulp might open doors for new therapeutic clinical strategies or improvement of already existing treatment possibilities. For this reason, it is important to explore and understand the basic molecular mechanisms of molecular clocks in the dental pulp.

Tooth trauma can lead to disruption of vessels and blood supply in the dental pulp, resulting in hypoxic conditions. Also, microsurgical treatments, such as pulpotomy, result in a hypoxic defect site. Hypoxia mimetic agents have already been applied as tools to simulate hypoxic conditions in vitro in dental pulp‐derived cells 13, 14. Interestingly, the hypoxia mimetic agent L‐mimosine (L‐MIM) and hypoxia are also used to synchronize cells, which is important for studies on gene expression oscillations. They both provide synchronisation by cell cycle arrest 15, 16. Hypoxia signalling in cells is mainly transmitted by hypoxia‐inducible factor (HIF)‐1*α*. Correlations between this transcription factor and circadian clock oscillation have been proposed 17 and could also play a role in the following investigated effects. Echinomycin is a widely used inhibitor of HIF‐1*α* signalling but has not yet been applied in analysis of the circadian clock 18, 19.

In this pilot study, we aimed to ascertain if human dental pulp‐derived cells (DPC) produce core circadian clock components at mRNA and protein levels and if this production is modulated by the hypoxia mimetic agent L‐MIM or hypoxia, alone or in combination with echinomycin. We tested our hypothesis in a traditional two‐dimensional (2D) monolayer model and in a three‐dimensional (3D) spheroid model (which mimics the in‐vivo environment more closely).

## Material and methods

### Cell isolation and culture conditions

Dental pulp‐derived cells from three different donors were harvested at the University Clinic of Dentistry, Medical University of Vienna (Vienna, Austria), as described before 14. The study protocol was approved by the Ethics Committee of the Medical University of Vienna. Extracted teeth without signs of inflammation are collected if informed consent is given by the patient. Pieces of extracted dental pulp tissue were cultured in alpha‐minimum essential medium (*α*MEM; Thermo Fisher Scientific, Waltham, MA, USA), containing 10% fetal bovine serum (Thermo Fisher Scientific), 100 U/ml of penicillin G, 100 mg/ml of streptomycin and 2.5 mg/ml of amphotericin B (Thermo Fisher Scientific). The heterogeneous cell population that grows out from these tissue pieces is referred to as DPC. Cells are either stored in liquid nitrogen or cultured at 37°C in 5% CO_2_ and 95% atmospheric moisture for experiments. All cell culture work was conducted under sterile conditions. Cell viability and differentiation potential under the experimental conditions used has already been shown in previous publications 14, 20.

### 2D cell culture

The 2D monolayer cultures were created by seeding 50,000 cells/cm^2^, from three different donors, into six‐well plates (TPP Techno Plastic Products, Trasadingen, SH, Switzerland). Experiments with this set‐up were performed twice. Then, 24 h after seeding, cells were treated with either 1 mM L‐MIM (Sigma‐Aldrich, St Louis, MO, USA) or hypoxia. The dose of L‐MIM was chosen based on the results of previous in‐vitro studies 13, 14. Hypoxia was induced as previously described, with minor modifications 21. In brief, DPC from three donors were seeded in six‐well plates (TPP Techno Plastic Products) and placed in a BD GasPak EZ Pouch system (Becton Dickinson, Franklin Lakes, NJ, USA). Cells without treatment under normoxic conditions served as control. In another set‐up, cells from two different donors were additionally treated with 1 *μ*M echinomycin alone or together with 1 mM L‐MIM or hypoxia. Experiments were performed twice. Dose‐finding experiments with echinomycin in DPC revealed 1 *μ*M as the most suitable dose, being in the range of previously reported doses 22, 23. For time course experiments, DPC were synchronized by serum‐starvation for 24 h. Afterwards, cells were treated with 1 mM L‐MIM or hypoxia and samples for mRNA or protein analysis were harvested every 4 h over a 48‐h period.

### 3D cell culture

To create 3D spheroid cell cultures of DPC we used 3D Petri Dishes (MicroTissues, Providence, RI, USA). Dishes for small spheroids were covered with 2% agarose (Sigma‐Aldrich) to produce moulds with 35 circular recesses. These moulds were incubated in cell culture medium (Thermo Fisher Scientific). Afterwards, the medium‐soaked moulds were transferred to 24‐well plates (TPP Techno Plastic Products) where they were filled with a 75 *μ*l suspension of 547,500 DPC derived from three different donors and covered with cell‐culture medium as described in the manufacturer's protocol. These DPC were subjected to the same treatments as the 2D cell cultures for 24 h. Experiments were performed twice.

### mRNA analysis

After the treatment period, total RNA was isolated from DPC of both culture models using the RNeasy Plus Mini Kit (Qiagen, Hilden, Germany), according to the protocol of the manufacturer. cDNA synthesis was carried out in a Primus 96 advanced Gradient thermal cycler (PEQLAB Biotechnologie, Erlangen, Germany) with a High Capacity cDNA Reverse Transcription Kit (Applied Biosystems, Carlsbad, CA, USA) and diluted as recommended by the manufacturer for quantitative PCR (qPCR). cDNA was amplified using TaqMan Real‐Time PCR Master Mix (Thermo Fisher Scientific) and TaqMan assays (Thermo Fisher Scientific) for *CLOCK* (Hs00231857_m1), *BMAL1* (Hs00154147_m1), *CRY1* (Hs00172734_m1), *CRY2* (Hs00323654_m1), *PER1* (Hs01092603_m1), *PER2* (Hs00256143_m1), and *PER3* (Hs00213466_m1). Three reference genes were evaluated, of which glyceraldehyde‐3‐phosphate dehydrogenase (*GAPDH*) (Hs02758991_g1) was determined as the most suitable in the experimental set‐up used. Quantitative PCR was performed with a StepOnePlus System (Thermo Fisher Scientific). mRNA levels were calculated using the ΔΔ*C*_t_ method, relative to *GAPDH* levels.

### Protein analysis

After the treatment period, total protein was isolated from DPC of both culture models to prepare samples for western blotting using the Laemmli Sample Buffer (Bio‐Rad Laboratories Vienna, Vienna, Austria) according to the manufacturer's description. The bicinchoninic acid (BCA) assay (Thermo Fisher Scientific) was performed according to the manufacturer's protocol to determine total protein concentration. Then, 1.2 mg total protein of each sample were separated by SDS‐PAGE. The following primary antibodies (Thermo Fisher Scientific) were used to detect the target proteins: anti‐CLOCK (PA5‐14532), anti‐CRY1 (PA5‐13124), anti‐CRY2 (PA5‐13125), and anti‐PER3 (PA5‐38634). Anti‐GAPDH (MA5‐15738) was used as reference antibody. These target proteins were chosen for western blotting based on the results of the qPCR. The primary antibody was detected with an appropriate secondary antibody using chemiluminescence detection with a ChemiDoc MP (Bio‐Rad Laboratories). For the time course experiments the band volume intensity was measured using the Image Lab software (Bio‐Rad Laboratories). Data are presented relative to the GAPDH control and normalized to the mean of the bands.

### Alkaline phosphatase staining

Monolayer DPC were cultured in differentiation medium consisting of *α*MEM (Thermo Fisher Scientific) containing 20% fetal calf serum (Thermo Fisher Scientific), antibiotics (Thermo Fisher Scientific), 10 mM *β*‐glycerophosphate (Sigma‐Aldrich), 50 mM L‐ascorbic acid (Sigma‐Aldrich), and dexamethasone (Sigma‐Aldrich). Dental pulp‐derived cells were fixed with formalin (Sigma‐Aldrich) at 1, 7 or 14 d after seeding and stained with alkaline phosphatase substrate (Sigma‐Aldrich) 24. Photographic and microscopic images at 100‐fold magnification were taken at each time point.

### Alizarin red staining

Dental pulp‐derived cell monolayer cultures in differentiation medium were fixed after 14 d with paraformaldehyde (Sigma‐Aldrich) and stained with an Alizarin Red (Sigma‐Aldrich). Photographic and microscopic images were taken at 100‐fold magnification.

### Statistical analysis

Statistical analysis was performed with IBM SPSS Statistics Version 23 (IBM, Armonk, NY, USA), using the Kruskal–Wallis test and the Mann–Whitney *U*‐test with Bonferroni correction. The level of significance was set at *P *<* *0.05. The null hypothesis tested was that there is no difference between the control group and the treated groups (L‐MIM or hypoxia).

## Results

### Core clock mRNA levels in 2D monolayer cultures of DPC in the presence of L‐MIM and hypoxia

Our results show that all seven core clock genes were expressed in DPC 2D monolayer cultures. The level of mRNA expressed by these clock genes relative to that of *GAPDH* was as follows (mean ± SD): *CLOCK* (0.0021 ± 0.0014), *BMAL1* (0.0002 ± 0.0002), *CRY1* (0.0018 ± 0.0026), *CRY2* (0.0060 ± 0.0079), *PER1* (0.0070 ± 0.0094), *PER2* (0.0012 ± 0.0015) and *PER3* (0.0033 ± 0.0040).

**Figure 1:**
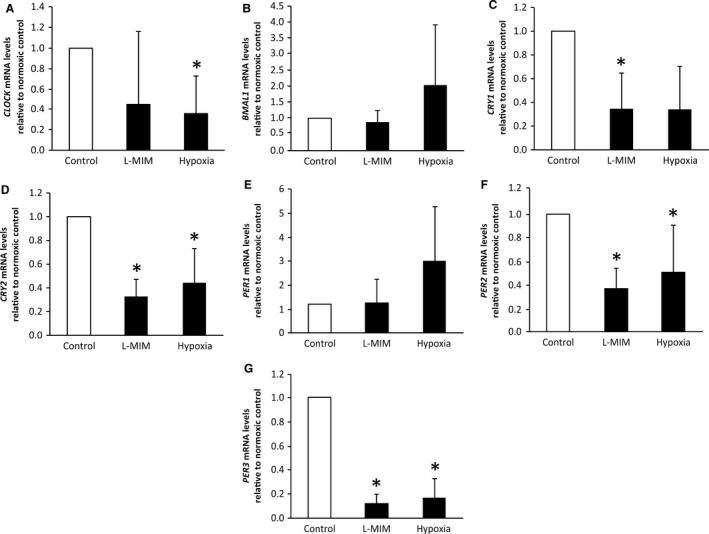
Effect of L‐mimosine (L‐MIM) and hypoxia on core clock gene mRNA levels in two‐dimensional (2D) monolayer cultures of dental pulp‐derived cells (DPC). The DPC in 2D monolayer cultures were treated with L‐MIM or hypoxia for 24 h. mRNA levels of the genes circadian locomotor output cycles kaput (*CLOCK*) (A), aryl hydrocarbon receptor nuclear translocator‐like protein 1 (*BMAL1*) (B), cryptochrome circadian regulator 1 (*CRY1*) (C) and 2 (*CRY2*) (D) and period circadian regulator 1 (*PER1*) (E), 2 (*PER2*) (F) and 3 (*PER3*) (G) were quantified by quantitative PCR (qPCR). Glyceraldehyde‐3‐phosphate dehydrogenase (*GAPDH*) was used as the reference gene. Bars represent mean + SD, relative to the normoxic control (white bar). Experiments were conducted twice with DPC from three different donors (*n *=* *6). *P <* *0.05.

In the DPC 2D monolayer culture, L‐MIM treatment significantly downregulated expression of mRNA for *CRY1* (*P *=* *0.004, *n *=* *6), *CRY2* (*P *=* *0.016, *n *=* *6), *PER2* (*P *=* *0.016, *n *=* *6) and *PER3* (*P *=* *0.016, *n *=* *6) relative to *GAPDH* and the normoxic control (Fig. 1). Expression of mRNA for *CLOCK* (*P *=* *0.170, *n *=* *6), *BMAL1* (*P *=* *0.788, *n *=* *6) and *PER1* (*P *=* *0.170, *n *=* *6) was not modulated significantly but showed a trend to decrease upon treatment with L‐MIM. Hypoxia significantly downregulated relative mRNA expression of *CLOCK* (*P *=* *0.004, *n *=* *6), *CRY2* (*P = 0.032*,* n *=* *6), *PER2* (*P *=* *0.016, *n *=* *6) and *PER3* (*P *=* *0.016, *n *=* *6) genes (Fig. 1). The data show a trend for upregulation of *BMAL1* (*P *=* *1.000, *n *=* *6) and *PER1* (*P *=* *0.788, *n *=* *6) and a trend for downregulation of *CRY1* (*P *=* *0.170, *n *=* *6), relative to mRNA levels after treatment with hypoxia, which did not reach significance.

### Core clock mRNA levels in 2D monolayer cultures of DPC in the presence of echinomycin

Echinomycin alone under normoxic conditions significantly downregulated production of mRNA for *BMAL1* (*P *=* *0.016, *n *=* *5), *CRY1* (*P *=* *0.016, *n *=* *5), *PER1* (*P *=* *0.016, *n *=* *5) and *PER2* (*P *=* *0.016, *n *=* *5) (Table 1), but also showed the same trend in the other genes. Echinomycin, used together with L‐MIM, significantly downregulated production of mRNA for *BMAL1* (*P *=* *0.016, *n *=* *5) compared with treatment with L‐MIM only (Table 1). *CLOCK* (*P *=* *1.000, *n *=* *5), *CRY1* (*P *=* *0.302, *n *=* *5), *CRY2* (*P *=* *1.000, *n *=* *5), *PER1* (*P *=* *0.302, *n *=* *5), *PER2* (*P *=* *0.302, *n *=* *5) and *PER3* (*P *=* *1.000, *n *=* *5) showed no significant modulation upon treatment with echinomycin plus L‐MIM. Treatment with echinomycin together with hypoxia showed a significant downregulation of *BMAL1* (*P *=* *0.016, *n *=* *5), *PER1* (*P *=* *0.016, *n *=* *5), and *PER2* (*P *=* *0.016, *n *=* *5) production, while *CLOCK* (*P *=* *0.302, *n *=* *5), *CRY1* (*P *=* *0.302, *n *=* *5), *CRY2* (*P *=* *1.000, *n *=* *5) and *PER3* (*P *=* *1.000, *n *=* *5) were not significantly modulated compared with treatment with hypoxia alone. The specific levels relative to the respective controls without echinomycin can be found in Table 1.

**Table 1:** Effect of echinomycin alone or with L‐mimosine (L‐MIM) or hypoxia on expression of mRNA for core circadian clock genes

Gene	Echinomycin	Echinomycin + L‐MIM	Echinomycin + Hypoxia
*CLOCK*	0.894 ± 0.534	2.422 ± 2.015	1.223 ± 1.713
*BMAL1*	0.317 ± 0.243*	0.260 ± 0.151*	0.200 ± 0.090*
*CRY1*	0.231 ± 0.196*	0.728 ± 0.590	0.673 ± 0.475
*CRY2*	0.652 ± 0.603	2.013 ± 1.871	0.983 ± 0.815
*PER1*	0.411 ± 0.315*	0.503 ± 0.674	0.235 ± 0.344*
*PER2*	0.246 ± 0.254*	0.664 ± 0.477	0.335 ± 0.355*
*PER3*	0.369 ± 0.311	4.459 ± 5.481	2.076 ± 1.645

Dental pulp‐derived cells (DPC) in two‐dimensional (2D) monolayer cultures were treated with echinomycin, with or without L‐MIM or hypoxia, for 24 h. The levels of mRNA expressed for the genes circadian locomotor output cycles kaput (*CLOCK*)*,* aryl hydrocarbon receptor nuclear translocator‐like protein 1 (*BMAL1*)*,* cryptochrome circadian regulator 1 and 2 (*CRY1* and *CRY2*, respectively) and period circadian regulator 1–3 (*PER1, PER2* and *PER3*, respectively) were measured by quantitative PCR (qPCR). Glyceraldehyde‐3‐phosphate dehydrogenase (*GAPDH*) was used as the reference gene. Numbers represent mean mRNA level ± SD, relative to the respective control without echinomycin (not shown in the table). Experiments were conducted twice with DPC from at least two donors (*n *=* *5). *P < 0.05.

### Core clock mRNA levels in DPC spheroid cultures in the presence of L‐MIM and hypoxia

In line with the 2D monolayer cultures, all seven core clock genes were expressed in 3D spheroid cultures. The mRNA expression levels of these genes relative to *GAPDH* were as follows (mean ± SD): *CLOCK* (0.0003 ± 0.0002), *BMAL1* (0.0002 ± 0.0001), *CRY1* (0.0002 ± 0.0001), *CRY2* (0.0018 ± 0.0012), *PER1* (0.0054 ± 0.0048), *PER2* (0.0002 ± 0.0001), and *PER3* (0.0006 ± 0.0003).

**Figure 2:**
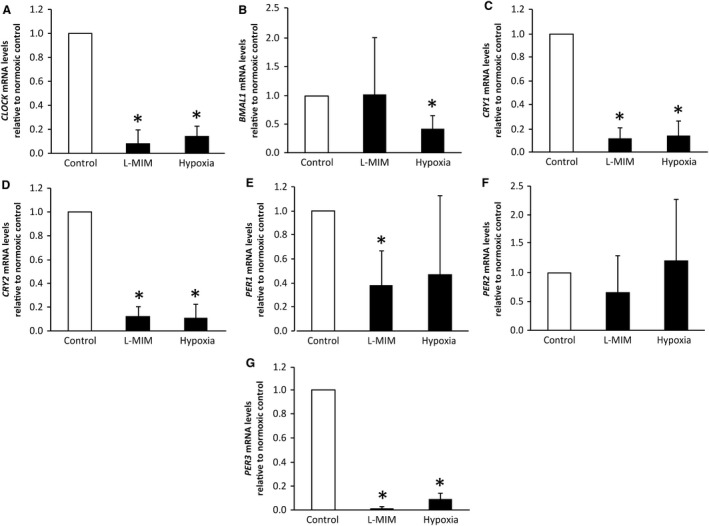
Effect of L‐mimosine (L‐MIM) and hypoxia on core clock gene mRNA levels in three‐dimensional (3D) spheroid cultures of dental pulp‐derived cells (DPC). The DPC in 3D spheroid cultures were treated with L‐MIM or hypoxia for 24 h. mRNA levels of the genes circadian locomotor output cycles kaput (*CLOCK*) (A), aryl hydrocarbon receptor nuclear translocator‐like protein 1 (*BMAL1*) (B), cryptochrome circadian regulator 1 (*CRY1*) (C) and 2 (*CRY2*) (D) and period circadian regulator 1 (*PER1*) (E), 2 (*PER2*) (F) and 3 (*PER3*) (G) were quantified by quantitative PCR (qPCR). Glyceraldehyde‐3‐phosphate dehydrogenase (*GAPDH*) was used as reference gene. Bars represent mean + SD, relative to the normoxic control (white bar). Experiments were conducted twice with DPC from three different donors (*n *=* *6). *P <* *0.05.

In the DPC 3D spheroid culture, the levels of mRNA for *CLOCK* (*P *=* *0.004, *n *=* *6), *CRY1* (*P *=* *0.004, *n *=* *6), *CRY2* (*P *=* *0.004, *n *=* *6), *PER1* (*P *=* *0.004, *n *=* *6) and *PER3* (*P *=* *0.004, *n *=* *6) were significantly decreased (*P *=* *0.788, *n *=* *6) relative to the normoxic control upon treatment with L‐MIM (Fig. 2). Relative expression of *PER2* (*P *=* *0.788, *n *=* *6) mRNA showed a trend to decrease but did not reach significance, while expression of *BMAL1* (*P *=* *0.788, *n *=* *6) mRNA did not show any modulation upon L‐MIM treatment. Hypoxia significantly downregulated relative mRNA levels of *CLOCK* (*P *=* *0.004, *n *=* *6), *BMAL1* (*P *=* *0.004, *n *=* *6), *CRY1* (*P *=* *0.004, *n *=* *6), *CRY2* (*P *=* *0.004, *n *=* *6) and *PER3* (*P *=* *0.004, *n *=* *6) genes (Fig. 2). Again, *PER1* relative mRNA levels showed a trend (*P *=* *0.170, *n *=* *6) for downregulation but did not reach significance and *PER2* mRNA levels were not modulated by hypoxia at all (*P *=* *0.788, *n *=* *6).

### Core clock protein levels in 2D monolayer and 3D spheroid cultures of DPC in the presence of L‐MIM, hypoxia and echinomycin

**Figure 3:**
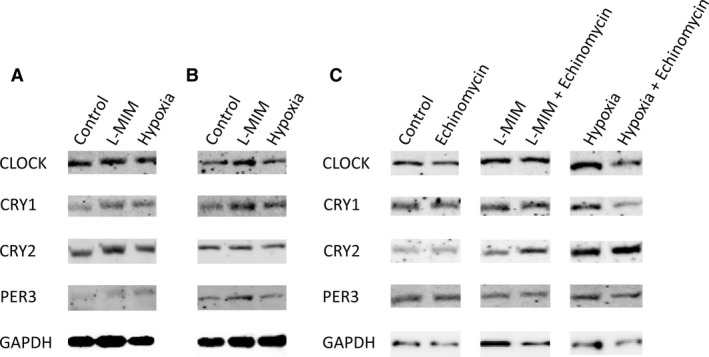
Effect of L‐mimosine (L‐MIM), hypoxia and echinomycin on the levels of core clock proteins in two‐dimensional (2D) monolayer and three‐dimensional (3D) spheroid cultures of dental pulp‐derived cells (DPC). The DPC in 2D monolayer cultures were treated with L‐MIM or hypoxia (A) and with L‐MIM or hypoxia in combination with echinomycin or with echinomycin alone (C) for 24 h. Further DPC were cultured in 3D spheroid cultures and treated with L‐MIM or hypoxia (B). The amounts of circadian locomotor output cycles kaput (CLOCK), cryptochrome circadian regulator 1 and 2 (CRY1 and CRY2, respectively) and period circadian regulator 3 (PER3) proteins produced were detected by western blotting. Glyceraldehyde‐3‐phosphate dehydrogenase (GAPDH) was used as reference protein. Experiments were conducted at least twice with DPC from two different donors (*n *=* *4).

Bands for all proteins were visible in control, L‐MIM and hypoxia samples of 2D monolayer (Fig. 3A) and 3D spheroid (Fig. 3B) DPC cultures. Also, samples treated with echinomycin, either alone or with L‐MIM or hypoxia, showed bands for the circadian clock proteins analysed (Fig. 3C). No visible modulation in band intensity was seen between the control and the different treatments (Fig. 3). Overall, band intensities differed between different cell donors (data not shown), but no effect of L‐MIM, hypoxia or echinomycin could be seen in samples from any of the donors. Our reference protein GAPDH was stably produced (Fig. 3).

### Core clock gene mRNA and protein levels in monolayer cultures of DPC under normoxia, L‐MIM and hypoxia over the 48‐h observation period

**Figure 4:**
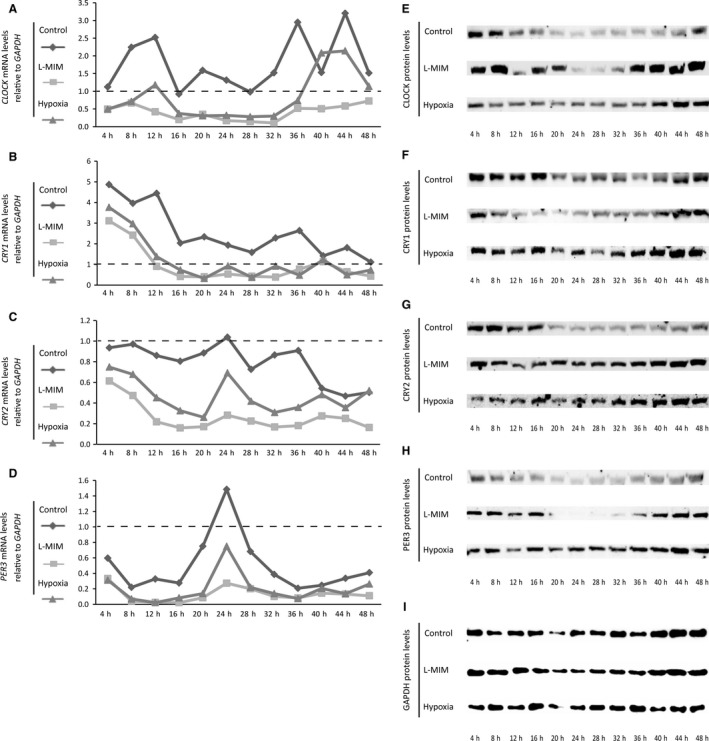
Core clock gene mRNA and protein levels under normoxia, L‐mimosine (L‐MIM) and hypoxia change during the observation period. Dental pulp‐derived cells (DPC) in two‐dimensional (2D) monolayer cultures were serum‐starved and afterwards treated with L‐MIM or hypoxia. mRNA (A‐D) and protein (E‐I) levels of circadian locomotor output cycles kaput (CLOCK), cryptochrome circadian regulator 1 and 2 (CRY1 and CRY2, respectively) and period circadian regulator 3 (PER3) were measured in a 4‐h interval over 48 h by quantitative PCR (qPCR) and western blotting, respectively. mRNA levels are displayed relative to glyceraldehyde‐3‐phosphate dehydrogenase (*GAPDH*) and 0 h after serum starvation (A‐D). GAPDH was used as reference protein (E‐I). Experiments were conducted at least twice with DPC from two different donors (*n *=* *4).

Analysis at mRNA level oscillations of core clock genes (Fig. 4A–D) showed that overall the relative expression level maxima and minima of the control group were higher than those of L‐MIM or hypoxia groups. The lowest maxima and minima were found in the L‐MIM group, which also showed the flattest profile (Fig. 4A–D). This effect was most dominant in *PER3*, where the time course of relative expression of all samples is similar (including the peak of the highest amplitude at 24 h) (Fig. 4D).

In line with our previous evaluation at 24 h, the results of protein expression do not reflect the modulation of mRNA expression by hypoxia and L‐MIM (Fig. 4E–I, Fig. S1). Overall core circadian clock protein levels also changed during the 48‐h observation period. Here, the hypoxia group showed the weakest changes over time.

To validate the differentiation capacity of DPC, the cells were cultured in differentiation medium. Alkaline phosphatase staining increased during culture of DPC for 14 d (Fig. S2A). After 14 d, matrix mineralization was observed upon staining DPC with Alizarin Red (Fig. S2B).

## Discussion

Core circadian clock genes set the pace for a variety of biological functions. Although the circadian clock has been proposed to be involved in tooth development, little is known about the role of core circadian clock genes and their regulation in the dental pulp 5. Thus, there is a need for studies that aim to reveal the relevance of molecular clocks in the dental pulp. Here, we have shown that the seven core circadian clock genes, namely *CLOCK*,* BMAL1*,* CRY1*,* CRY2*,* PER1*,* PER2* and *PER3*, are expressed in human cells of the dental pulp of permanent dentition in 2D monolayer and 3D spheroid cultures. Thereby, we provide the first indications for the components of a functional peripheral circadian clock in the dental pulp. Our data show that the effects of L‐MIM and hypoxia treatment are comparable, but not the same, in the two models. The effects in 3D culture are stronger than in 2D culture, which is important for future studies. While a myriad of valuable knowledge is based on in vitro research with 2D monolayer cell cultures, 3D cell cultures are a new approach that better reflect the in vivo situation. Here, our use of a 3D spheroid model of DPC is the first time that a 3D in vitro model has been used to study a circadian clock context. Spheroids are also widely used as in vitro disease models, especially for cancer 25. This is another relevant aspect because molecular clocks are known to be dysregulated in pathology 26.

A further important characteristic of the in vivo environment is that the dental pulp does not consist of one single cell type but is composed of different cell types and cells at a variety of differentiation states. Accordingly, we did not focus on a specific subpopulation but used heterogeneous cell populations generated from dental pulp tissue explant cultures 13, 14, 27, 28. These cell populations consist not only of fibroblastic cells, but also contain cells with stem‐cell characteristics and have the capacity to differentiate into the odontoblastic/osteoblastic lineage 14, 28. In this publication, we have shown that when DPC are cultured in differentiation medium for 14 d, alkaline phosphatase and matrix mineralization, both markers of differentiation into the odontoblastic/osteoblastic lineage, increase (Fig. S1). In a previous publication, these and further characteristic factors were also shown at mRNA level 14. Future studies should evaluate the link between circadian core clock gene oscillation and the expression of stem cell markers and how hypoxia or hypoxia mimetic agents can modulate the interaction. We used human DPC from different donors in 2D monolayer cultures and 3D spheroid cultures in vitro and therefore the results cannot be generalized to other cells, tissues, species or even the in vivo situation, but they can serve as a primer for future investigations on the role of the circadian clock in the dental pulp.

A link between the circadian clock and the hypoxic signalling pathway and their interaction with each other in both directions has previously been proposed 29. Hypoxia plays a role in disrupting circadian rhythm in certain cancer models 30 and can increase PER1 and CLOCK expression in murine brains 31. As an explanation for these findings, regulation by HIF‐1*α*, a major transcription factor of the hypoxic pathway, has often been suggested. It has been reported that HIF‐1*α* directly influences the circadian rhythm 17, 32 and also cooperates with clock genes to regulate downstream expression of target genes 33, 34. L‐mimosine 13, 18, 19, 35, 36 and hypoxia 18, 37 are also known to stabilize HIF‐1*α*; therefore, it was reasonable to analyse a potential HIF‐1*α* dependency in our experimental set‐up with the *HIF‐1α* signaling pathway inhibitor echinomycin. The results of these experiments show that, under normoxic conditions, echinomycin alone can have an effect on the mRNA levels of core clock genes. Significant modulation that resulted from application of echinomycin together with L‐MIM or hypoxia suggests at least a partial involvement of HIF‐1*α*.

This study showed that L‐MIM and hypoxia downregulate different core circadian clock genes in different cell culture models. The most prominent changes were found in *CLOCK*,* CRY1*,* CRY2* and *PER3* mRNA levels. For this reason, we decided to analyse the levels of CLOCK, CRY1, CRY2 and PER3 protein in another step and found these proteins to be produced in both cell culture models and in all cell culture conditions. In contrast to our mRNA data, protein data did not show any clear modulation by L‐MIM, hypoxia or echinomycin, implying that mRNA and protein production do not parallel each other. It is possible that the effects seen at mRNA level could also be observed at protein levels but at different time points because of the different time frames of transcription and translation. To show a more detailed pattern of the modulations observed at mRNA level, core circadian clock genes and proteins in DPC monolayer cultures were measured after cell synchronisation over 48 h. The mRNA results of these time course experiments suggest that the modulatory effects of L‐MIM and hypoxia might be based on a change in expression amplitude rather than a shift in expression oscillation. Although a modulation was seen over time of CLOCK, CRY1, CRY2 and PER3 protein levels, it did not reflect the mRNA data. It might therefore be of relevance for future experiments to include an entrainment phase in which cells should not only be synchronized but additionally entrained to a circadian 24‐h rhythm (e.g. with a light stimulus). Nevertheless, these results also show that choice of treatment and sample harvest timing should be carefully chosen and considered to be different for mRNA and protein measurements. Interestingly, the levels of PER3 protein seem to differ from the levels of *PER3* mRNA in the control and L‐MIM samples, in that PER3 protein tends to have a weaker signal around 24 h whereas the levels of *PER3* mRNA have their highest peak around 24 h.

The results of this study have kick‐started a new field of research in endodontics with potential innovative clinical applications. In other fields of medicine, chronotherapy and chronopharmacology are already being practiced, meaning that a specific timing for therapy or drug delivery is determined to reach maximal effects 38. Dental pulp sensibility is suggested to have different intensities depending on the time of day; this could be relevant for endodontology 39. In future, it has to be evaluated if different therapies in dentistry should be performed at a specific time point during the day in patients. The effects of a variety of lasers and lights used in dentistry 40, 41, 42, as well as wound‐healing applications using light therapy, could be based on the mechanism of the circadian clock driven by light 42.

## Conflicts of interest

The authors deny any conflict of interest.

## Supporting information


**Fig. S1.** Core clock protein levels under normoxia, L‐MIM and hypoxia change during the 48‐h observation period.
**Fig. S2.** Alkaline phosphatase and matrix mineralisation were detectable in DPC cultured in osteoblast differentiation medium.
