# Supplementary material for: Core circadian clock gene expression in human dental pulp‐derived cells in response to L‐mimosine, hypoxia and echinomycin
Source: Eur J Oral Sci. 2018 Jul 13;126(4):263–71. doi: 10.1111/eos.12535 (PMC6585758; doi:10.1111/eos.12535)
Supplement: Supplementary file 1 — Fig. S1. Core clock protein levels under normoxia, L‐MIM and hypoxia change during the 48‐h observation period. Fig. S2. Alkaline phosphatase and matrix mineralisation were detectable in DPC cultured in osteoblast differentiation medium. [file EOS-126-263-s001.pdf]

Supporting Information

Core circadian clock gene expression in human dental pulp-derived cells in response to L-mimosine, hypoxia and echinomycin

JANJIĆ K, KURZMANN C, MORITZ A, AGIS H

School of Dentistry, Medical University of Vienna
Austrian Cluster for Tissue Regeneration, Vienna, Austria

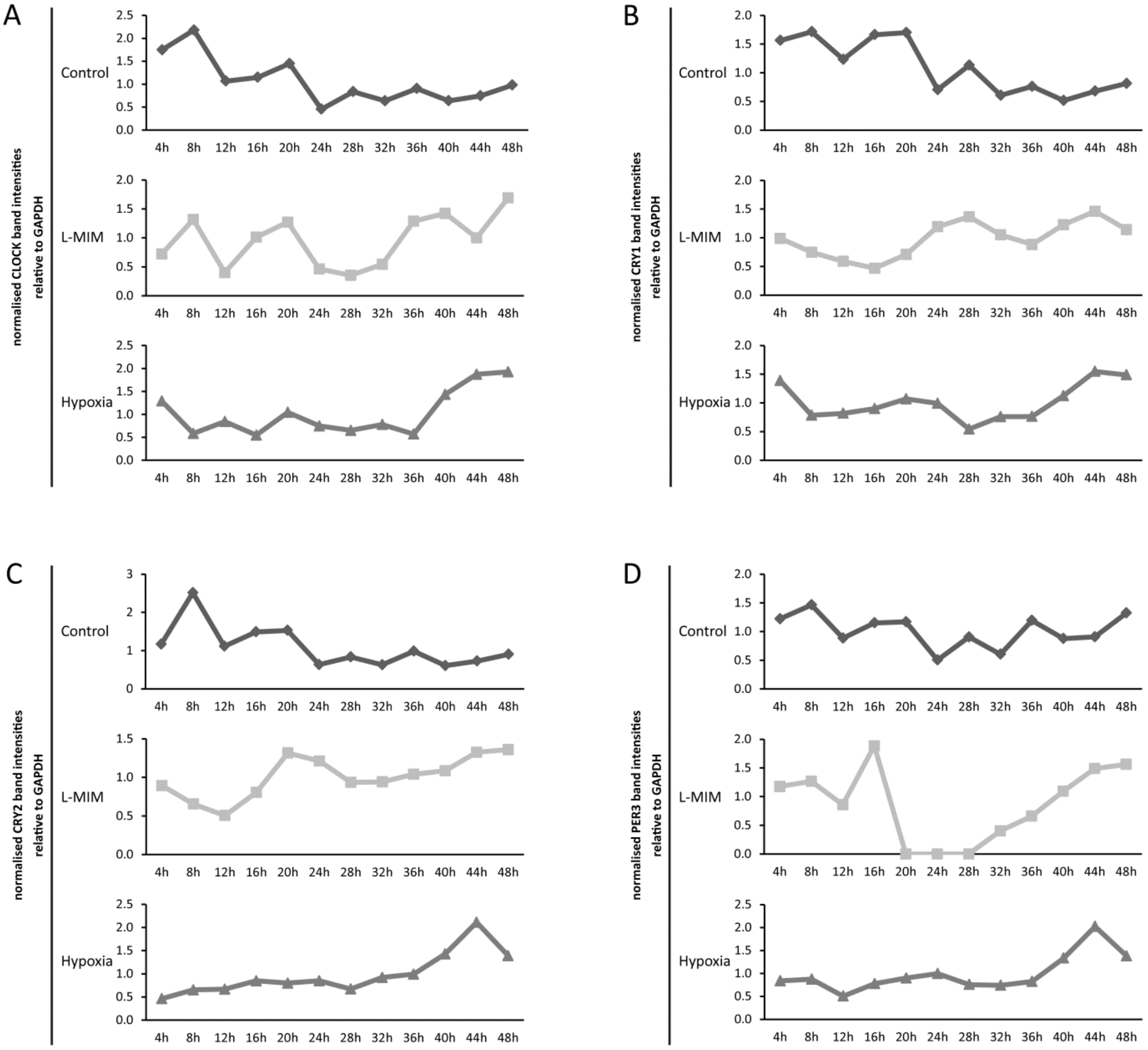

Figure S1: Core clock gene protein levels under normoxia, L-MIM and hypoxia change during the observation period. Dental pulp-derived cells (DPC) in 2D monolayer cultures were serum starved and afterwards treated with L-mimosine (L-MIM) or hypoxia. Protein levels of CLOCK, CRY1, CRY2 and PER3 were measured in a 4 h interval over 48 h by Western Blot (See also Fig. 4). Protein levels are displayed as band volume intensity relative to GAPDH and normalized to the mean band volume intensity of the respective proteins in the blot (A-D).

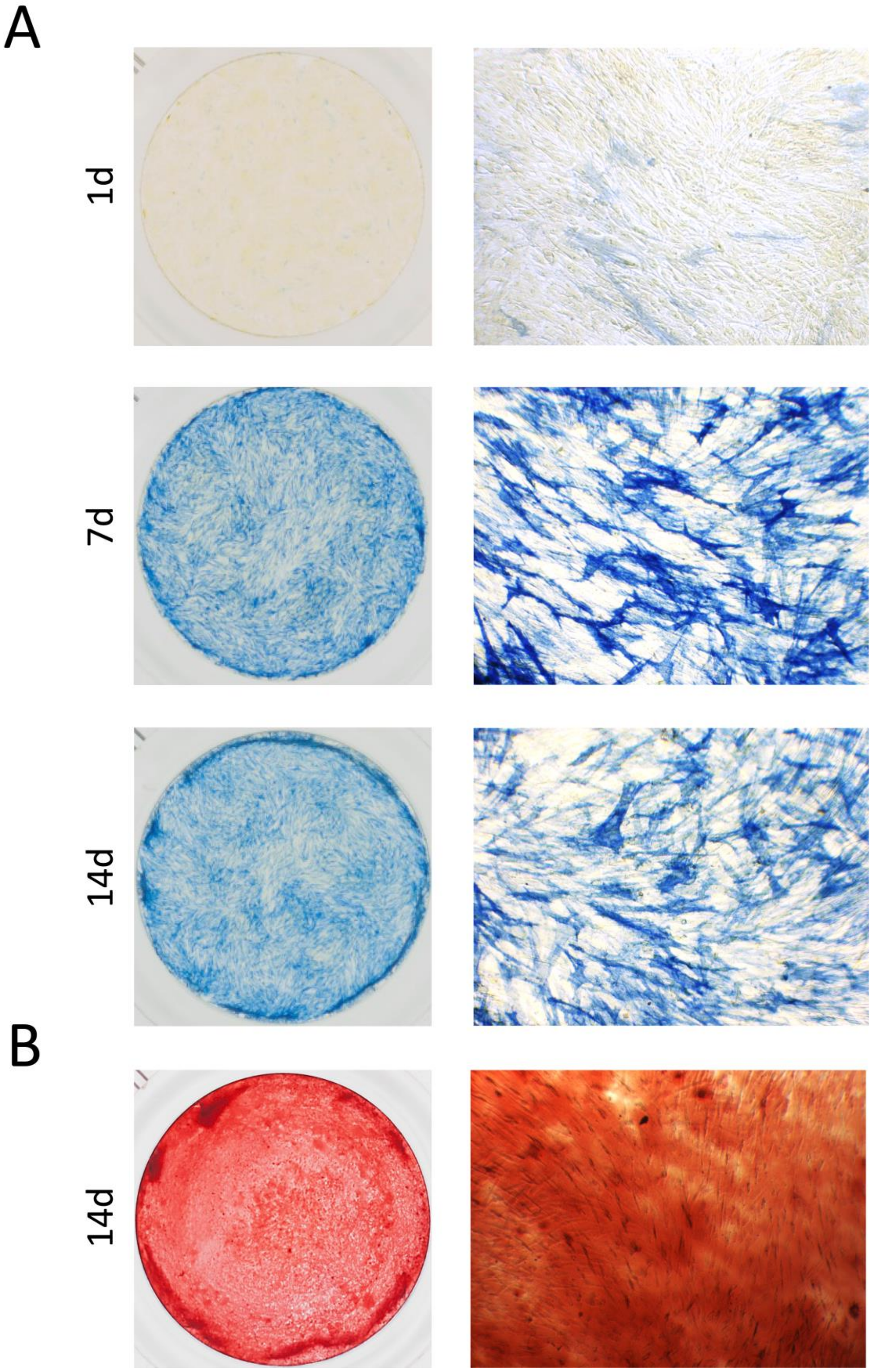

Figure S2: Detection of alkaline phosphatase and matrix mineralisation in DPC cultured in osteoblast differentiation medium. Dental pulp-derived cells (DPC) were cultured in osteoblast differentiation medium and stained with alkaline phosphatase (A) at 1d, 7d and 14d, and with alizarin red for matrix mineralization (B) 14 d after seeding. Photographic (left) and microscopic (right) images were taken at 100-fold magnification.
